# Different *Bacteroides* Species Colonise Human and Chicken Intestinal Tract

**DOI:** 10.3390/microorganisms8101483

**Published:** 2020-09-27

**Authors:** Miloslava Kollarcikova, Marcela Faldynova, Jitka Matiasovicova, Eva Jahodarova, Tereza Kubasova, Zuzana Seidlerova, Vladimir Babak, Petra Videnska, Alois Cizek, Ivan Rychlik

**Affiliations:** 1 Veterinary Research Institute, 62100 Brno, Czech Republic; kollarcikova@vri.cz (M.K.); faldynova@vri.cz (M.F.); matiasovicova@vri.cz (J.M.); evajahodarova@gmail.com (E.J.); kubasova@vri.cz (T.K.); Seidlerova@vri.cz (Z.S.); babak@vri.cz (V.B.); 2 Faculty of Science, Masaryk’s University, 62500 Brno, Czech Republic; petra.videnska@recetox.muni.cz; 3 Department of Infectious Diseases and Microbiology, Faculty of Veterinary Medicine, University of Veterinary and Pharmaceutical Sciences, 61242 Brno, Czech Republic; cizeka@vfu.cz

**Keywords:** microbiota, pentose cycle, glutamate decarboxylase, microbiome, chicken, human, caecum, *Bacteroides*

## Abstract

Bacteroidaceae are common gut microbiota members in all warm-blooded animals. However, if Bacteroidaceae are to be used as probiotics, the species selected for different hosts should reflect the natural distribution. In this study, we therefore evaluated host adaptation of bacterial species belonging to the family Bacteroidaceae. *B. dorei*, *B. uniformis*, *B. xylanisolvens*, *B. ovatus, B. clarus*, *B. thetaiotaomicron* and *B. vulgatus* represented human-adapted species while *B. gallinaceum*, *B. caecigallinarum*, *B. mediterraneensis*, *B. caecicola*, *M. massiliensis*, *B. plebeius* and *B. coprocola* were commonly detected in chicken but not human gut microbiota. There were 29 genes which were present in all human-adapted *Bacteroides* but absent from the genomes of all chicken isolates, and these included genes required for the pentose cycle and glutamate or histidine metabolism. These genes were expressed during an in vitro competitive assay, in which human-adapted *Bacteroides* species overgrew the chicken-adapted isolates. Not a single gene specific for the chicken-adapted species was found. Instead, chicken-adapted species exhibited signs of frequent horizontal gene transfer, of KUP, *linA* and *sugE* genes in particular. The differences in host adaptation should be considered when the new generation of probiotics for humans or chickens is designed.

## 1. Introduction

Bacteria that form the gut microbiota of warm-blooded animals belong to numerous phyla, but representatives of two phyla—Gram-negative Bacteroidetes and Gram-positive Firmicutes—commonly form over 90% of all microbiota in healthy adults [1,2,3,4,5,6]. Bacteroidetes have a higher potential to interact with their host and degrade complex polysaccharides [7,8,9], and their metabolism commonly produces acetate, propionate and succinate [10,11]. Firmicutes preferentially degrade oligosaccharides [7] or ferment metabolic byproducts such as lactate or acetate into butyrate [10,11]. The most common representatives of Firmicutes belong to families Lactobacillaceae, Erysipelotrichaceae, Lachnospiraceae, Ruminococcaceae, Clostridiaceae and Veillonellaceae. Isolates belonging to phylum Bacteroidetes are classified mainly into families Rikenellaceae, Porphyromonadaceae, Prevotellaceae and Bacteroidaceae [12]. Out of these families, Bacteroidaceae with genera *Bacteroides* or *Mediterranea* represent one of the most frequent Gram-negative colonisers of the distal intestinal tract [3,4]. Although the composition of gut microbiota down to family level follows similar patterns in omnivorous, warm-blooded animals, including humans, at the lowest taxonomic levels a certain degree of host adaptation is anticipated. We never specifically stressed in our previous studies on chicken microbiota, but we repeatedly observed that 16S rRNA sequence of chicken *Megasphaera* sp. is only 94.5% similar to human *Megasphaera elsdenii*. Similarly, chicken *Faecalibacterium* isolates are 96% similar to *Faecalibacterium prausnitzii* from human. Although host adaptation can be expected, this has never been experimentally addressed. Understanding microbiota adaptation to its host and vice versa is important also for the following reasons. First, looking at genetic differences in human- or chicken-adapted species belonging to the same genus may help with understanding specific functions of particular bacterial species for their host. Secondly, if considering novel types of probiotics, these should reflect host adaptation, since millions of years of evolution and natural selection likely selected the best performing metaorganism consisting of microbiota and host.

We have recently initiated systematic culturing of chicken gut anaerobes including their characterisation by whole genome sequencing and use of these strains as probiotics in chickens [3,7,13]. Since selective conditions for individual microbiota members are not known, the knowledge of whole genomic sequence was used for the identification of species-specific genes and design of specific PCRs to follow different strains in intestinal tract of chickens when these were tested as potential probiotics. Interestingly, the use of these PCRs for the detection of different *Bacteroides* species showed that not all of them were equally abundant in chickens. When we later rather accidentally tested a limited number of human stool samples using *Bacteroides*-specificprimers, we noticed that *Bacteroides* species common in the chickens were rare in humans and, conversely, species less abundant in the chickens were common in human stool samples. This was quite unexpected because all Bacteroidaceae isolates in our laboratory collection originated from chickens and, despite this, some of them appeared as apparently non-native to chickens. It is possible that young chickens, due to the absence of contact between hen and chicks in commercial production, might be colonised by non-native microbiota members, of human personnel origin. If this was the case, using caecal contents of young chickens as a source for culture of bacterial species best adapted for young chickens [7] may represent a misleading idea. False conclusions with such an approach can be further “amplified” by picking of individual colonies what may end up in enrichment of culture collection by non-native chicken microbiota members. However, these were only hypotheses which had to be confirmed experimentally. The major aim of this study therefore was to clearly determine human- and chicken-adapted *Bacteroides* species and to identify genes specific for human- and chicken-adapted *Bacteroides* species. In addition, we also tested mutual competition between human- and chicken-adapted *Bacteroides* species and decided whether the genes specific for human- or chicken-adapted *Bacteroides* species can explain their competitiveness during in vitro growth.

## 2. Materials and Methods 

### 2.1. Selection of Target Species

Host adaptation of 14 different species belonging to family Bacteroidaceae was evaluated in this study. Target species included *B. dorei*, *B. clarus*, *B. uniformis*, *B. xylanisolvens*, *B. ovatus*, *B. thetaiotaomicron*, *B. vulgatus*, *B. gallinaceum*, *B. caecigallinarum*, *B. plebeius*, *B. coprocola*, *B. caecicola*, *B. mediterraneensis* and *Mediterranea massiliensis* characterised by whole genomic sequencing previously [7]. 

### 2.2. Design of Species-Specific Primers

To evaluate distribution of the above-listed *Bacteroides* species in human or chicken samples, we designed species-specific real-time PCRs. First, we identified 3 genes (only 2 genes in *B. vulgatus*) which were present only in the genome of the target species but were absent from the genomes of all other species using their functional annotation. Three primer pairs for the detection of each of 14 species were then designed to three different genes and tested using template DNA purified from three different mixtures of strains with known composition (see Table S1 for primer sequences). The first mixture consisted of *B. dorei*, *B. plebeius*, *B. caecigallinarum* and *M. massilliensis*, the second mixture consisted of *B. clarus*, *B. uniformis*, *B. xylanisolvens*, *B. ovatus* and *B. salanitronis* and the last mixture consisted of *B. plebeius* and *B. coprocola*. Primer pairs specific for *B. thetaiotaomicron*, *B. vulgatus*, *B. caecicola* and *B. mediterraneensis* were tested using pure DNA of these species. Out of 3 different primer pairs, the primer pair providing the lowest Ct value was selected for each species, and this subset of 14 species-specific primer pairs was finally evaluated in PCRs using pure DNA from all 14 species as a template (Table S2) as well as checked against GeneBank entries.

### 2.3. PCR Conditions

Real-time PCR was performed in 3 µL volumes in 384-well microplates using QuantiTect SYBR Green PCR Master Mix (Qiagen, Hilden, Germany) and a Nanodrop pipetting station (Innovadyne, Santa Rosa, CA, United States) for dispensing PCR mixtures. PCR and signal detection were performed with a LightCycler II (Roche) with an initial denaturation at 95 °C for 15 min followed by 40 cycles of denaturation at 95 °C for 20 s, primer annealing at 61 °C for 30 s and extension at 72 °C for 30 s. Each sample was subjected to real-time PCR in duplicate. Amplification of eubacterial 16S rRNA genes was used as a reference to determine the total amount of bacterial DNA in each sample. The Ct values of PCRs specific to different *Bacteroides* species were normalized to an average Ct value of 16S rRNA gene amplification (ΔCt) and the percentage representation of each *Bacteroides* species in a given sample was finally calculated using the formula 100 × 1/2^−ΔCt^.

### 2.4. Human and Chicken Samples

In total, 70 human faecal samples originating from healthy individuals, 68 caecal samples of chickens older than 1 month, hereafter called hens, 24 caecal samples of chickens younger than 1 month, 21 caecal samples from turkey and 20 faecal samples from pigs were collected (Table S3). The chicken’s age was taken into consideration due to the delayed appearance of *Bacteroides* in the caeca of commercially raised chickens [4]. DNA from all samples was purified using QIAamp Stool kit according to the manufacturer’s instructions (Qiagen) and was used as a template in SybrGreen real-time PCR (Qiagen) as described above. 

### 2.5. Clustering of Bacteroides Species

Multiple sequence alignment was performed in Mafft v6.240 with Q-INS-I algorithm [14]. 16S rRNA gene sequences of all 14 *Bacteroides* species and an additional 21 *Bacteroides* species characterised previously [7] were rooted to the outgroup *Prevotella ruminicola* CHR7 16S rRNA sequence (GenBank accession number MH708239). Bayesian inference was estimated by BEAST 1.8.1 [15] with the HKY + I + G model, applying a strict clock (rate = 1). The analysis was run for 10,000,000 generations, with a sampling frequency of 10,000. Tracer v.1.7. [16] was used for checking ESS values and run convergence plateau. Maximum credibility tree was estimated using TreeAnnotator v1.10 [15] after discarding the initial 30% of trees. Maximum likelihood analysis was performed in IQ-TREE v1.6.12 [17]. The substitution model was identified using ModelFinder [18] implemented in IQ-TREE (option -m TEST). ML analysis was tested using ultrafast bootstrapping with 10,000 replicates [19].

### 2.6. Genome Analysis, Identification of Human- or Chicken-Specific Genes

RAST assigned gene names of all 14 *Bacteroides* species were used for the comparison of numbers of genes belonging to gene families with the same biological functions. RAST was used also for sequence-based comparison of genes present either in human- or chicken-adapted *Bacteroides* species [20]. In this case, one of the strains was selected as a reference and sequences of its genes were compared with gene sequences of the remaining isolates. Genes with a similarity lower than 50% were considered as unrelated while genes with a similarity higher than 99% were considered as subjected to recent horizontal transfer.

### 2.7. Anaerobic Culture, Growth Media and In Vitro Growth Competition

The isolates were subcultured in anaerobic atmospheric conditions consisting of 85% N_2_, 10% CO_2_ and 5% H_2_ on BHI agar at 37 °C for 3 days. Prior to in vitro growth competition, the isolates were resuspended in pre-reduced PRAS dilution blank (0.1 g magnesium sulfate heptahydrate, 0.2 g monobasic potassium phosphate, 0.2 g potassium chloride, 1.15 g dibasic sodium phosphate, 3.0 g sodium chloride, 1.0 g sodium thioglycolate, 0.5 g L-cysteine, 1000 mL distilled water; final pH 7.5 +/− 0.2 at 25 °C) to OD (600 nm) = 1, and equal volumes of all isolates were mixed to form the inoculum. An aliquot of the inoculum was taken for real-time PCR to determine strain ratios prior growth competition, and 500 µL of the mixture was used for the inoculation of 20 mL of BHI broth or BHI broth supplemented with mucin, cellulose, starch and pectin in 200-mL plastic bottles. All the supplements were used at 0.5% concentration as described previously [21]. After three-day incubation under the anaerobic conditions, 500 µL of culture was used for inoculation of new batches of nutrient broths. The subculturing was repeated three times so that together with the first culture, we obtained bacterial mass from 4 consecutive batches. The bacterial mass from each broth and subculture was collected from 1.5 mL of medium by 1 min centrifugation at 13,000× *g* and the pellets were frozen at −20 °C until use.

### 2.8. Protein Mass Spectrometry

Bacterial pellets were lysed in 1 mL of Tri Reagent and processed following the manufacturer’s recommendations (MRC). Obtained proteins were further processed by the filter aided sample preparation method [22] and analysed using an UltiMate 3000 RSLCnano liquid chromatograph (Dionex) connected to a LTQ-Orbitrap Velos Pro mass spectrometer (Thermo Scientific, Waltham, MA, USA). Chromatographic separation was performed in three technical replicates using an EASY-Spray C18 separation column (15 cm × 75 µm, 3 µm particles, Thermo Scientific) and a two-hour-long acetonitrile gradient.

High resolution (30,000 FWHM at 400 m/z) MS spectra were acquired for the 390–1700 m/z interval in an Orbitrap analyser (Thermo Scientific) with an AGC target value of 1 × 106 ions and maximal injection time of 100 ms. Low resolution MS/MS spectra were acquired in Linear Ion Trap in a data-dependent manner, and the top 10 precursors exceeding a threshold of 10,000 counts and having a charge state of +2 or +3 were isolated within a 2 Da window and fragmented using CID.

Raw data were analysed using Proteome Discoverer v.1.4 (Thermo Scientific). MS/MS spectra identification was performed by SEQUEST (Thermo Scientific) using isolate-specific protein database deduced from their known whole genomic sequence. Only peptides with a false discovery rate FDR ≤ 5% were used for protein identification. Peptide spectral matches (PSM) counts were finally used as a quantitative parameter for each protein in each sample. Data are available via ProteomeXchange with identifier PXD020954.

### 2.9. Statistics

Abundances of individual *Bacteroides* species in human, older chicken and younger chicken samples were compared by the Kruskal–Wallis test followed by Dunn’s post hoc test using STATISTICA 13.2 software (Dell, Inc., Tulsa, OK, USA). A Mann–Whitney test was used for the comparison of genome sizes and the comparison of numbers of particular genes in genomes of human or chicken *Bacteroides* species. Differences with *p* < 0.05 were considered as significant in all analyses.

### 2.10. Ethics Approval and Consent to Participate

All human participants provided informed written consent and the study was approved by the European Longitudinal Study of Pregnancy and Childhood Steering Committee, Masaryk University. The handling of animals in the study was performed in accordance with current Czech legislation (Animal Protection and Welfare Act No. 246/1992 Coll. of the Government of the Czech Republic). The specific experiments were approved by the Ethics Committee of the Veterinary Research Institute followed by the Committee for Animal Welfare of the Ministry of Agriculture of the Czech Republic (permit number MZe1922). Authors also declare that not a single chicken has been sacrificed specifically for the purpose of this study and DNA purified from all chicken samples originated from previous studies of authors’ team when verbal consent of sample providers was obtained.

## 3. Results

### 3.1. Distribution of Bacteroides Species in Chicken and Human Samples

**Figure 1:**
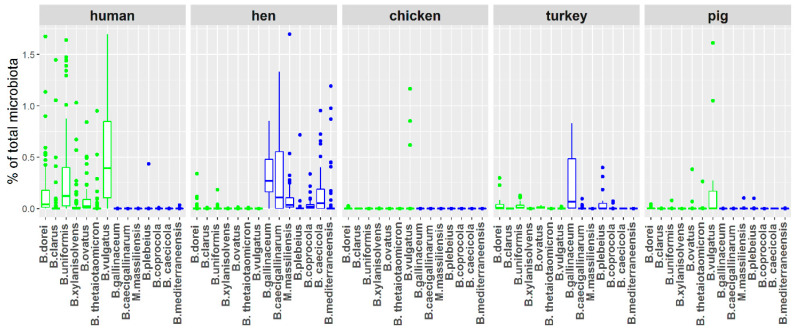
Host specificity of *Bacteroides* species. *B. dorei*, *B. uniformis*, B. thetaiotaomicron, *B. vulgatus*, *B. xylanisolvens* and *B. ovatus* (green boxes) were significantly more abundant in human samples than in hen or chicken samples (Kruskal–Wallis test followed by Dunn’s test, *p* < 0.01) and these therefore represent human-adapted species. *B. salanitronis*, *B. caecigallinarum*, *M. massiliensis*, *B. plebeius*, *B. caecicola*, *B. mediterraneensis* and *B. coprocola* (blue boxes) were significantly more abundant in hen than in human samples, and these therefore represent chicken-adapted species.

All the designed primer pairs exhibited species specificity although GenBank BLAST comparison indicated that *B. ovatus* primers may detect some of *B. xylanisolvens* strains. When the set of specific PCRs was used for the detection and quantification of *Bacteroides* species in human faecal and chicken caecal samples, host-dependent distribution was observed. *B. clarus, B. dorei, B. uniformis, B. xylanisolvens, B. ovatus, B. thetaiotaomicron* and *B. vulgatus* were significantly more abundant in human samples than in hen or chicken samples (Figure 1) and these species were therefore considered as human adapted. On the other hand, *B. gallinaceum, B. mediterraneensis, B. caecicola, B. salanitronis, B. caecigallinarum, M. massiliensis, B. plebeius* and *B. coprocola* were significantly more abundant in hen samples than in human or chicken samples (Figure 1, Table S3). We therefore considered these species as adapted to adult hens. To determine whether the detected *Bacteroides* species were human and chicken adapted, or rather mammals or avian adapted, we tested their presence also in pig faecal samples and turkey caecal samples. *B. clarus, B. dorei, B. uniformis, B. xylanisolvens* and *B. ovatus* were strictly human adapted while *B. thetaiotaomicron* and *B. vulgatus* were common also in pigs. Similarly, *B. caecicola*, *B. salanitronis*, *B. caecigallinarum*, *M. massiliensis* and *B. coprocola* were strictly adapted to chickens. *B. gallinaceum* and *B. mediterraneensis* were common both in chickens and turkeys and *B. plebeius* was even more frequent in turkey than in chicken gut microbiota (Figure 1 and Table S3).

### 3.2. Clustering of Chicken- and Human-Adapted Bacteroides Species

**Figure 2:**
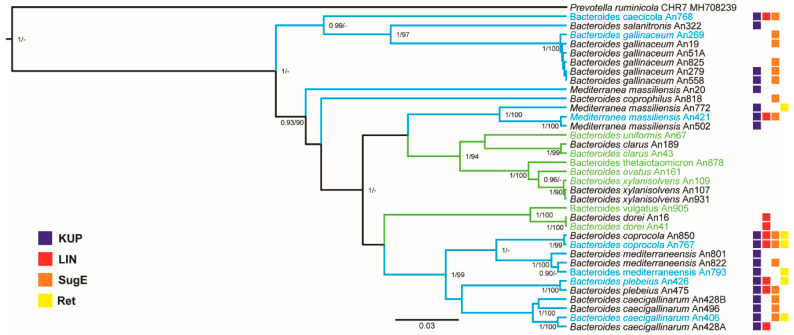
A phylogenetic reconstruction of 35 Bacteroidaceae isolates based on 16S rRNA sequence using Bayesian inference. Supports at the nodes represent Bayesian inference and maximum likelihood, respectively. Only posterior probabilities >0.90 and bootstrap values >90 are shown. Clades in blue or green represent chicken- or human-adapted *Bacteriodes* species, respectively. Isolates in blue represent chicken-adapted species selected for genomic comparisons with human isolates. Isolates in green represent human-adapted species selected for genomic comparisons with chicken isolates. Presence of *kup*, *linA*, *sugE* and *ret* genes in each genome is shown by blue, red, green and yellow squares, respectively. For alternative alignment using *rpoB* nucleotide sequence see Figure S1.

Next, we used sequences from our previous study [7] and clustered all available *Bacteroides* species based on their whole length 16S rRNA gene sequences. Chicken- and human-adapted *Bacteroides* species formed separated lineages. Of human isolates, *B. clarus*, *B. uniformis*, *B. xylanisolvens*, *B. thetaiotaomicron* and *B. ovatus* belonged to the same lineage and *B. dorei* and *B. vulgatus* clustered separately (Figure 2). Chicken-adapted *Bacteroides* species formed three major lineages. The first was formed by *B. caecicola*, *B. salanitronis* and *B. gallinaceum*, the second by *B. plebeius*, *B. coprocola*, *B. mediterraneensis* and *B. caecigallinarum* and the third by *M. massiliensis* (Figure 2).

### 3.3. Genome Differences between Human- and Chicken-Adapted Bacteroides Species

**Figure 3:**
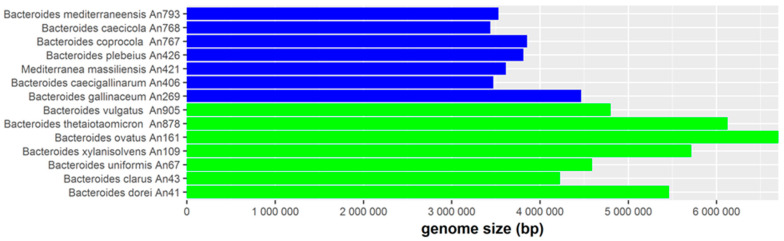
Genome size of chicken (blue columns) and human (green columns) adapted *Bacteroides* species. Human-adapted *Bacteroides* species exhibited a significantly higher genome size than chicken-adapted species (Mann–Whitney test, *p* < 0.05).

The separation of human- and chicken-adapted *Bacteroides* species into different lineages based on their 16S rRNA sequence prompted us to compare their genomes. Genomes of chicken-adapted *Bacteroides* species were significantly smaller in size (ranging from 3.43 Mb to 4.46 Mb) than genomes of human-adapted *Bacteroides* species (ranging from 4.23 Mb to 6.70 Mb, see Figure 3).

**Figure 4:**
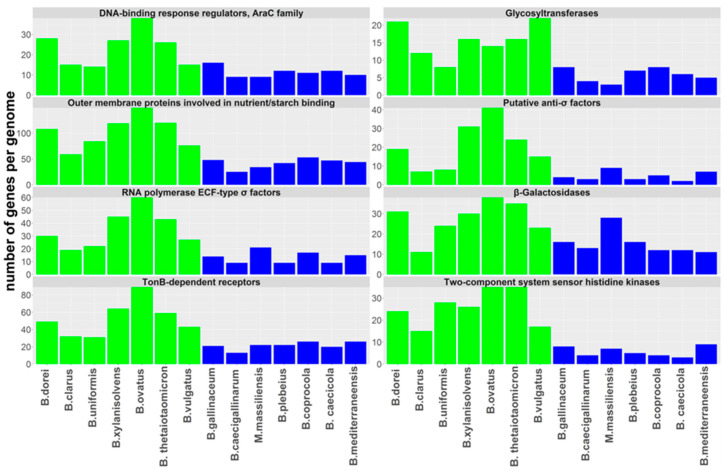
Number of genes belonging to specific gene families in human- and chicken-adapted *Bacteroides* species. Eight gene families exhibited a significantly higher number of genes in the genomes of human-adapted *Bacteroides* isolates compared to those from chickens (Mann–Whitney test, *p* < 0.05). Human-adapted *Bacteroides* species (green columns) harbored significantly more genes coding for proteins sensing extracellular conditions and polysaccharide binding and transport than chicken-adapted *Bacteroides* species (blue columns). See Table S4 for the list of genes belonging to all gene families in human- and chicken-adapted *Bacteroides* species.

Next, we tested whether there were any biological processes or genes specific for chicken- or human-adapted *Bacteroides* species. Firstly, we performed a comparison based on predicted gene function, i.e., not strictly dependent on gene sequence. This comparison was performed for gene families comprising at least 10 different genes of similar function per genome. Genes of not a single gene family were more abundant in the genomes of chicken-adapted *Bacteroides* species. On the other hand, there were eight gene families, genes of which were significantly more abundant in the genomes of human-adapted *Bacteroides*. These included four gene families involved in saccharide metabolism incl. TonB-dependent receptors, outer membrane proteins involved in nutrient/starch, β-galactosidases and glycosyltransferases, and additional four gene families coding for regulatory proteins including a two-component system sensor histidine kinases, ECF-type sigma factor RNA polymerases, DNA-binding response regulators of AraC family and putative anti-sigma factors (Figure 4).

### 3.4. Identification of Genes Specific for Human- or Chicken-Adapted Bacteroides Species

The second analysis was based on gene sequence similarities and was aimed at the identification of individual genes which were present in all chicken-adapted *Bacteroides* species but were absent in all human isolates, and vice versa. There were 29 different genes present in all human-adapted *Bacteroides* but absent from the genomes of chicken isolates (Table 1). Of these, all human-adapted *Bacteroides* species encoded proteins for histidine catabolism, i.e., formiminotetrahydrofolate cyclodeaminase, imidazolonepropionase, glutamate formiminotransferase and urocanate hydratase. All human-adapted *Bacteroides* species also encoded proteins for glutamate metabolism, i.e., glutamate/γ-aminobutyrate antiporter, glutamate decarboxylase and glutamine synthetase. Finally, genes coding for enzymes of the pentose cycle such as decarboxylating 6-phosphogluconate dehydrogenase, glucose-6-phosphate 1-dehydrogenase and transaldolase were also present only in human *Bacteroides* species.

Unlike human-adapted *Bacteroides* species, there was not a single gene which was present in all seven chicken-adapted *Bacteroides* species and absent in all human-adapted species. However, when performing this analysis, we noticed that there were genes among the chicken-adapted *Bacteroides* species which were present in the genomes of three to five species (i.e., not in all seven chicken-adapted species) but which exhibited 99–100% similarity. These genes coded for retron-type RNA-directed DNA polymerase, quaternary ammonium compound-resistance protein SugE, Kup system potassium uptake protein, putative macrolide efflux pump, lincosamide nucleotidyltransferase LinA, acetyltransferase GNAT family, FIG00631715: hypothetical protein and mobilization protein. Of these, putative macrolide efflux pump, lincosamide nucleotidyltransferase LinA, acetyltransferase GNAT family, FIG00631715: hypothetical protein and mobilization protein formed a conserved genomic island of 5 genes. Since slightly relaxed criteria were used for the selection of these genes as chicken *Bacteroides* species adapted, we next analysed the distribution of these genes in all *Bacteroides* species which we have sequenced so far (Figure 1). The gene for Kup system potassium uptake protein was absent from the genomes of all isolates belonging to the two clades of human-adapted species but was present in 19 out of 24 chicken-adapted isolates. Lincosamide nucleotidyltransferase genomic island was present in 2 out of 11 human-adapted strains and in 7 out of 24 chicken-adapted isolates (Figure 2). Retron-type RNA-directed DNA polymerase was present in six chicken but no human-adapted isolate and gene for quaternary ammonium compound-resistance *sugE* was present in 15 chicken but no human-adapted isolate.

### 3.5. Do Host Adaptation and Differences in Gene Content Affect Bacteroides Competition?

**Figure 5:**
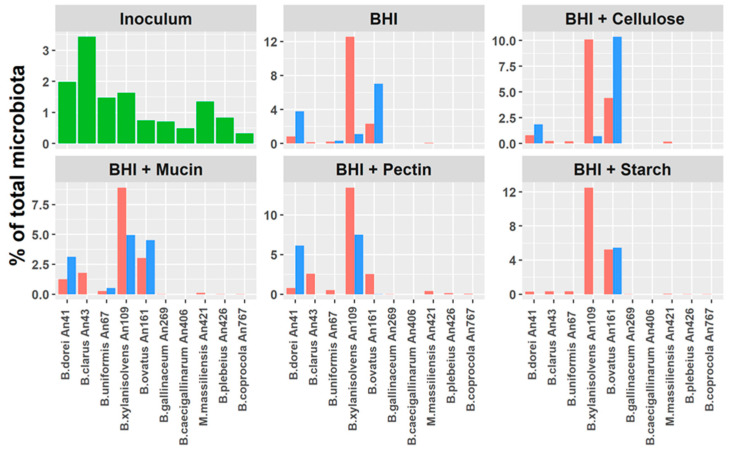
Real-time PCR quantification of *Bacteroides* species after co-culture in differently supplemented BHI broths. Human-adapted species overgrew the chicken-adapted species immediately after the first subculture. After four passages, the microbial community was formed mainly by *B. xylanisolvens, B. ovatus* and *B. dorei. B. xylanisolvens* and *B. dorei* were positively selected by pectin whilst starch and cellulose positively selected for *B. ovatus*. Supplementation with mucin was neutral in selection among *B. xylanisolvens*, *B. ovatus* and *B. dorei* and, instead, partially supported the growth of *B. uniformis*. Green columns—composition of the inoculum, blue columns—abundance after first subculture, red columns—abundance after 4th subculture. Data originate from a single experiment.

Whether the differences in gene content affect *Bacteroides* competition was tested by co-culture in BHI broth supplemented with mucin, cellulose, starch or pectin. Only 10 strains, 5 human and 5 chicken adapted, were included in this experiment. The abundance of all inoculated strains was determined by a real-time PCR specific for each isolate. The major difference among the strains was that each of the human-adapted *Bacteroides* species grew faster in any of the tested media than any of the chicken-adapted isolates (Figure 5). Among human isolates, *B. clarus* and *B. uniformis* grew more slowly than *B. xylanisolvens*, *B. ovatus* and *B. dorei*. Different growth rates restricted the comparison of growth preferences only to *B. xylanisolvens*, *B. ovatus* and *B. dorei*. Of these, non-supplemented BHI negatively affected the growth of *B. xylanisolvens*. The addition of mucin did not select for any of the three compared strains but weakly supported the growth of *B. uniformis*. Supplementation of BHI broth with pectin positively selected for *B. xylanisolvens* and *B. dorei* whilst cellulose and starch supplementation supported the growth of *B. ovatus* (Figure 5).

### 3.6. Are Genes Specific for Human Bacteroides Species Expressed during Co-Culture In Vitro?

Total protein was purified from bacterial mixtures obtained after growth in BHI supplemented media and subjected to protein mass spectrometry. Out of the 29 genes specific for human-adapted *Bacteroides* species, 25 were expressed by at least one strain in at least one of the tested media (Table 1 and Table S5). However, not all the proteins were expressed at the same level. Proteins commonly and highly expressed in human-adapted *Bacteroides* included glutamate decarboxylase (1.05% average abundance of all proteins), 6-phosphogluconate dehydrogenase (0.21%), pirin (0.11%), transaldolase (0.08%), glucose-6-phosphate 1-dehydrogenase (0.09%), endonuclease IV (0.04%) and nitroreductase family protein (0.04%).

## 4. Discussion

Bacteroidaceae are common gut microbiota members in all warm-blooded animals and, not surprisingly, their broad distribution results in them being considered as probiotics [23,24]. However, if Bacteroidaceae are to be used as probiotics, their selection should reflect their natural distribution in different hosts. In this study, we therefore tested host adaptation of 14 bacterial species from family Bacteroidaceae. *B. dorei*, *B. clarus*, *B. uniformis*, *B. xylanisolvens*, *B. ovatus*, *B. thetaiotaomicron* and *B. vulgatus* represented human-adapted species while *B. salanitronis*, *B. caecigallinarum*, *M. massiliensis*, *B. plebeius*, *B. coprocola*, *B. caecicola* and *B. mediterraneensis* were specific for chickens. A certain degree of host adaptation could be expected as *B. dorei*, *B. uniformis*, *B. xylanisolvens* and *B. ovatus* were reported as common to human gut microbiota [8,9,25,26] and *B. plebeius* and *B. caecigallinarum* are common in chickens [27,28]. However, the extent and contrast of the host adaptation was unexpected, since representatives of 12 species were originally isolated from chickens [7] and *B. thetaiotaomicron* and *B. vulgatus* were originally cultured from pig faeces. It is unlikely that the contrast was caused by comparing chicken caecal samples with human faecal samples since conditions in the chicken caecum resemble conditions in distal parts of human digestive tract the most. Moreover, both human- and chicken-adapted *Bacteroides* species efficiently colonise the caecum of chicks during the first week of life [3,13], which apparently contradicts host adaptation. This contradiction can be explained by the fact that the human-adapted *Bacteroides* species can temporarily colonise chickens and are likely introduced to chicken flocks by contact with humans. Despite this, these species do not persist in the chicken intestinal tract over the whole life of chickens and are replaced by chicken-adapted species. Similarly, chicken-adapted species can temporarily colonise humans, since *B. plebeius* was also recorded in human microbiota [29,30] and *M. massiliensis* was originally cultured from a human sample [31]. Our observations are similar to the host adaptation observed in other bacterial species like serotypes of *Salmonella enterica* [32]. We also cannot exclude that there can be *Bacteroides* species broadly distributed among different hosts due to variability in pangenome. In other words, it was quite risky to reduce presence of particular species to a single gene quantification since there are species in which their pangenome dominate core genome [33] and individual strains may exhibit different ecological adaptations [34]. Despite this, data in Figure 1 clearly present that at least the species which we detected follow host adaptation. This way of thinking also corroborates experiments with germ-free mice colonised with microbiota from different sources, but colonised the best with microbiota from conventional mice in comparison to microbiota from human, zebrafish, and termite guts [35].

Host adaptation correlated with differences at the genomic level. Using 16S rRNA sequences, human- and chicken-adapted species formed different lineages. The genomes of human-adapted isolates were larger, thus allowing these isolates to encode more genes for polysaccharides uptake (Figure 4). Only human-adapted isolates encoded and expressed genes for the pentose cycle or degradation of glutamate and histidine. Whether this corroborates differences in diet, human diet being enriched for proteins in comparison to chicken diet enriched for saccharides of plant origin, is not known. On the other hand, the genomes of chicken-adapted isolates exhibited signs of recent horizontal gene transfer. As this was recorded after analysing a very small number of compared genomes, horizontal transfer must be common and frequent. The gene coding for Kup system potassium uptake protein and the presence of quaternary ammonium compound-resistance protein SugE was highly characteristic for chicken isolates. The presence of quaternary ammonium compound-resistance protein SugE in the genomes of chicken-adapted *Bacteroides* species must be a consequence of positive selection of this resistance due to the use of antimicrobials in poultry production. On the other hand, wide distribution of *linA* among chicken *Bacteroides* sp. was rather surprising, since lincosamides are not antibiotics of the first choice in poultry.

Differences between human- and chicken-adapted isolates were observed also during in vitro growth. The fact that some species overgrew the others was not unexpected since *B. ovatus* was already reported as belonging among rapidly multiplying species from human microbiota [36]. Rather unexpected was that all human-adapted species outgrew all the chicken-adapted species. Since BHI medium used for the growth competition is rich in peptides and amino acids, highly expressed glutamate decarboxylase in all human-adapted isolates could be at least partially responsible for the difference. Faster multiplication of human-adapted isolates could be caused also by the expression of the pentose cycle and the availability of ribose and deoxyribose for nucleotide biosynthesis, DNA replication and RNA transcription. Differential multiplication of human and chicken *Bacteroides* species can also be a consequence of adaptation to the different physiology of the intestinal tracts of humans and chickens. The distal intestinal tract in humans is represented by the colon with continuous peristalsis. On the other hand, the colon in chickens is very short and the major part of distal intestinal tract in chickens is represented by the caecum. The caecum in chickens is filled with digesta from the ileum which is then fermented in the caecum for around 12 h [37]. Conditions in the human colon therefore resemble a continuous culture while growth in the chicken caecum is similar to batch culture. Faster multiplication is therefore required for survival in the human colon with its continuous flow, while slow-growing species might be better adapted to the batch-like culture conditions in the chicken caecum.

## 5. Conclusions

In this study, we pointed towards a high level of adaptation of *Bacteroides* species in either chicken or human hosts, which are associated with differences in genome structure and basal metabolism. Though we refer to the compared isolates as chicken and human adapted, equally important might be the difference between *Bacteroides* lineages itself. Future studies may reveal that, besides adaptation to continuous flow or batch-like fermentation, the adaptation might be for avian hosts or mammals as we have shown by analysis of limited number of samples from turkeys and pigs. The adaptation may be also diet dependent and apparently chicken-adapted *Bacteroides* species may be of increased abundance in humans with a higher proportion of polysaccharides and fibre in their food [38]. In conclusion, isolates from young animals in particular should be critically evaluated for their host specificity and real probiotic potential especially when new generations of probiotics for humans or chickens are designed.

## Figures and Tables

**Table 1:** Genes present in all human-adapted *Bacteroides* species but absent from chicken-adapted *Bacteroides* species.

Gene	An41 *	An109	An161	An43	An67	An878	An905	N. expr.^#^	Abund. (%) ^$^
Glutamate decarboxylase	100	84.23	84.02	84.94	84.73	83.61	97.93	55	1.054
Glucose-6-phosphate 1-dehydrogenase	100	58.06	58.47	58.84	60.44	58.27	97.37	53	0.090
6-phosphogluconate dehydrogenase, decarboxylating	100	61.65	61.23	61.6	60.55	62.5	95.4	50	0.206
Pirin	100	58.12	67.52	59.4	56.84	66.67	92.74	47	0.109
Transaldolase	100	85.78	86.24	85.78	85.78	86.24	98.62	44	0.084
Nitroreductase family protein	100	64.29	63.78	60.71	61.73	66.33	95.02	32	0.039
Endonuclease IV	100	75.95	74.05	79.01	78.46	74.05	96.18	29	0.043
Osmosensitive K+ channel histidine kinase KdpD	100	77.13	76.6	78.49	80.65	78.07	98.93	26	0.015
Integral membrane protein	100	58.83	58.11	56.9	57.77	58.21	97.36	12	0.029
Glutamate/γ-aminobutyrate antiporter	100	83.05	83.47	80	80.62	83.47	98.12	12	0.029
Glutamine synthetase type I	100	83.8	83.8	81	82	86.44	97.98	10	0.073
YbbM seven transmembrane helix protein	100	74.24	73.48	73.21	69.81	74.62	98.87	9	0.018
probable uroporphyrin-III c-methyltransferase	100	58.47	60.17	56.78	61.02	57.27	97.46	5	0.009
FIG00936253: hypothetical protein	100	78.35	77.32	78.35	82.72	80.41	99.07	3	0.011
Imidazolonepropionase	100	70.05	70.39	65.78	65.53	69.86	98.55	3	0.006
Cell division protein MraZ	100	68.42	67.67	60.87	67.67	68.42	97.83	3	0.006
Formiminotetrahydrofolate cyclodeaminase	100	54.68	56.65	55.96	57.14	55.83	98.55	2	0.005
Urocanate hydratase	100	84.98	84.07	81.03	81.54	84.94	97.59	2	0.007
FIG00406657: hypothetical protein	100	61.92	61.67	59.71	58.54	60.93	97.31	2	0.007
Glutamate formiminotransferase	100	79.33	79.33	85.19	87.12	82.67	98.98	1	0.002
COG1272: Predicted membrane protein hemolysin III	100	58.69	58.69	55.29	54.81	59.81	98.58	1	0.002
Potassium-transporting ATPase A chain	100	85.74	85.92	85.74	85.21	85.21	99.12	1	0.007
Potassium-transporting ATPase C chain	100	77.13	76.6	81.82	79.03	77.25	96.83	1	0.024
D-alanyl-D-alanine dipeptidase	100	68.56	66.67	66.98	70.65	65.7	98.19	1	0.002
FIG00407539: hypothetical protein	100	68.12	70.44	56.59	59.02	67.79	95.69	n.e.	n.e.
Cold shock protein CspA	100	50	50	51.68	58.11	53.69	99.32	n.e.	n.e.
FIG00407264: hypothetical protein	100	70	68.82	63.74	62.35	68.82	99.42	n.e.	n.e.
FIG00412566: hypothetical protein	100	56.85	56.85	54.58	54.47	55.37	95.53	n.e.	n.e.
membrane protein ykgB	100	70.31	70.83	87.11	90.21	70.31	95.9	n.e.	n.e.

*—gene sequences of *B. dorei* (An41) were used as a reference to find the most similar genes in the genomes of the remaining human-adapted *Bacteroides* species. Numbers in the table show percentage similarity at amino acid level. ^#^—protein expression was determined for five human-adapted *Bacteroides* species in the inoculum and five different media after the first and fourth subculture. If the given protein was expressed by all isolates in the inoculum and in all tested media (as was the case for glutamate decarboxylase), the maximal value in this column would be 55. When the given protein was recorded only in some of the strains or only in some of the tested media, the value is correspondingly lower. n.e.—not expressed. ^$^—data in this column show average abundance of a given protein out of the total protein during in vitro growth.

## Supplementary Materials

The following files are available online at https://www.mdpi.com/2076-2607/8/10/1483/s1, Figure S1: Multiple alignment of *rpoB* gene sequence of 35 different *Bacteroides* isolates. Strains in blue and green represent chicken and human isolates initially used for the design of species-specific primers. Blue and green lineages represent chicken- or human-adapted species, respectively. Table S1: Sequences of species-specific primer pairs. Table S2: Specificity of selected primer pairs with target and non-target DNAs. Table S3: Origin of samples, year of isolation and abundance of tested *Bacteroides* species (in percentage out of total bacterial population determined by real time PCR). Table S4: Number of genes belonging to all gene families in human- and chicken-adapted Bacteroides species. Table S5: Abundance of individual proteins in percentage calculated from PSM counts.

## Abbreviations

KUP	K+ (potassium) uptake protein
BHI broth	Brain Heart Infusion broth
GNAT	Gcn5-related N-acetyltransferase
