# Supplementary material for: Different Bacteroides Species Colonise Human and Chicken Intestinal Tract
Source: Microorganisms. 2020 Sep 27;8(10):1483. doi: 10.3390/microorganisms8101483 (PMC7600693; doi:10.3390/microorganisms8101483)

Figure S1: Multiple alignment of *rpoB* gene sequence of 35 different *Bacteroides* isolates. Strains in blue and green represent chicken and human isolates initially used for the design of species-specific primers. Blue and green lineages represent chicken- or human-adapted species, respectively.

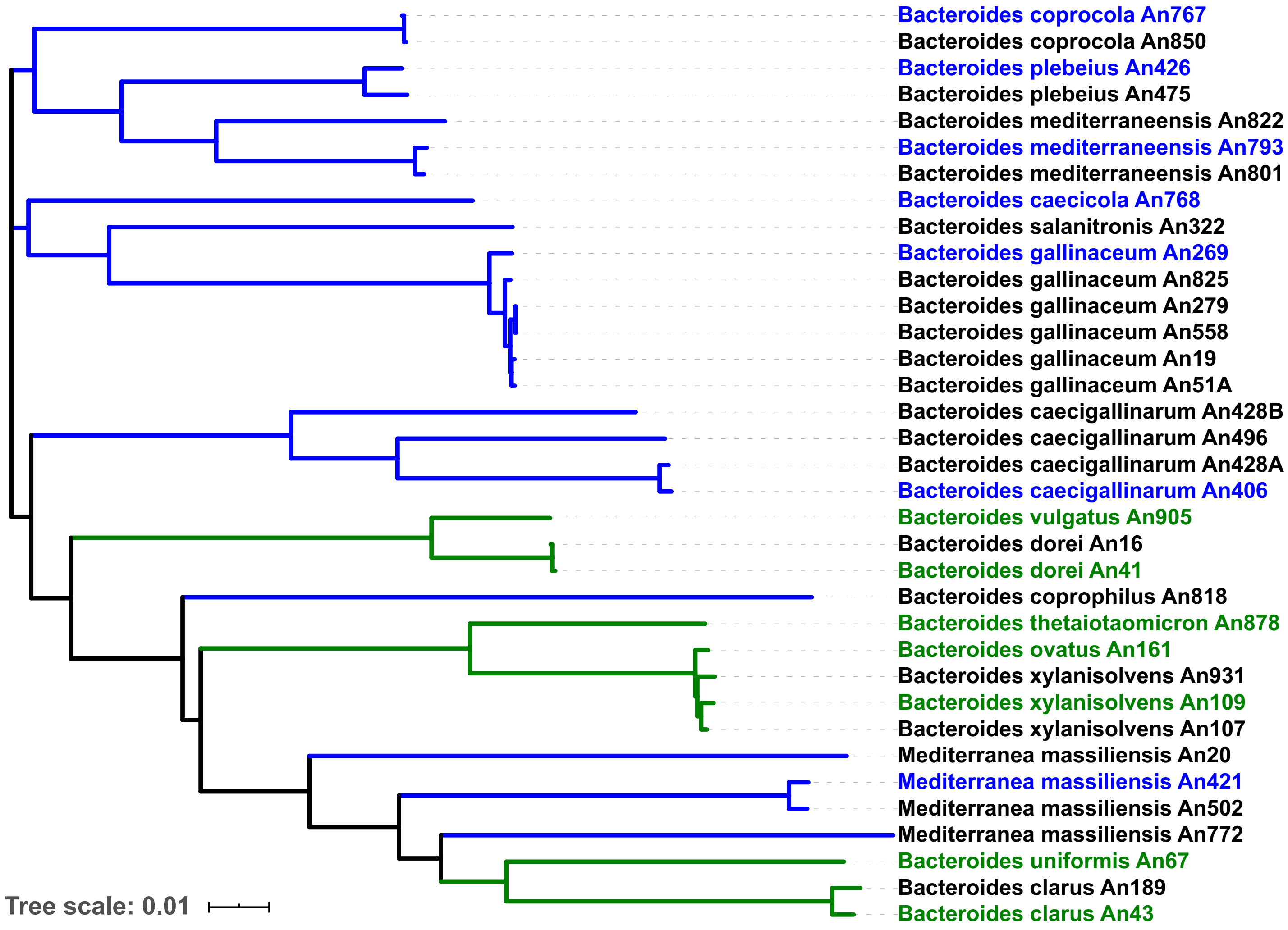

Supplement: Supplementary file 1 [file microorganisms-08-01483-s001.zip › Figure S1.pdf]
